# How different viruses perturb host cellular machinery via short linear motifs

**DOI:** 10.17179/excli2023-6328

**Published:** 2023-10-26

**Authors:** Sobia Idrees, Keshav Raj Paudel, Tayyaba Sadaf, Philip M. Hansbro

**Affiliations:** 1 School of Biotechnology and Biomolecular Sciences, University of New South Wales, Sydney, NSW, Australia; 2 Centre for Inflammation, Centenary Institute and the University of Technology Sydney, School of Life Sciences, Faculty of Science, Sydney, New South Wales, Australia

**Keywords:** virus, protein-protein interactions, short linear motifs, mimicry

## Abstract

The virus interacts with its hosts by developing protein-protein interactions. Most viruses employ protein interactions to imitate the host protein: A viral protein with the same amino acid sequence or structure as the host protein attaches to the host protein's binding partner and interferes with the host protein's pathways. Being opportunistic, viruses have evolved to manipulate host cellular mechanisms by mimicking short linear motifs. In this review, we shed light on the current understanding of mimicry *via *short linear motifs and focus on viral mimicry by genetically different viral subtypes by providing recent examples of mimicry evidence and how high-throughput methods can be a reliable source to study SLiM-mediated viral mimicry.

## Introduction

Most viruses use similar strategies to impact cellular circuits by imitating host patterns (Benedict et al., 2002[10]; Chaurushiya et al., 2012[13]; Davey et al., 2011[18]; Finlay and McFadden, 2006[35]). Most of the time, globular domains mediate protein interactions through interactions. It was once believed that only the proteome's globular domains and structured regions could mediate protein-protein interactions (PPIs). However, advances in proteomics have demonstrated that disordered regions with Short Linear Motifs (SLiMs) also significantly contribute to PPIs. Viral cells interact with host cellular proteins via SLiMs that resemble SLiMs in host cells. Several biological activities, such as PPIs, post-translational modifications, regulation, and cell compartment targeting, use SLiMs (Davey et al., 2012[19]; Neduva and Russell, 2005[81]). SLiMs, which are strong and rapidly evolving components found in viruses, cause the rewiring of the virus-host PPIs (vhPPIs) (Chemes et al., 2015[14]). Primarily, a SLiM that is sufficiently visible on a protein surface can govern protein stability; more generally, it can control ligand binding and targeting, which can manage various functions. The human proteome is about 30 % disordered (Garg et al., 2022[38]; Neduva and Russell, 2005[81]; Van Roey et al., 2014[109]). These intrinsically disordered regions (IDRs) and occasionally inaccessible loops inside their folded domains contain SLiMs that are evolutionarily changeable protein regions that can change with a single-point mutation (Neduva and Russell, 2005[81]; Van Roey et al., 2014[109]). IDRs have functional significance but still need extensive characterization (Diella et al., 2008[24]; Hornbeck et al., 2012[52]; Nguyen Ba et al., 2012[83]). 

In unrelated proteins, SLiMs may arise *de-novo* due to convergent evolution, further complicating our understanding of the interactome (Tompa and Csermely, 2004[107]). As per estimation, the human proteome has about 1 million SLiMs (Tompa et al., 2014[108]), explaining the complexity of the regulatory mechanisms of cells. SLiMs in pathogenic proteins are called mimicry motifs because they have similar (if not identical) amino acid composition and function to host SLiMs (Elkhaligy et al., 2021[31]). Many mimicry motifs exist in pathogens, particularly in attachment, penetration, and cytolysis proteins (Goswami et al., 2023[43]). One of the viruses' most well-known mimic motifs is PxxPxR (polyproline motif). This motif has been reported in non-structural 5A proteins (NS5A in hepatitis C virus and Nef protein (Nef in HIV type 1) and establishes interactions with the SH3 domains of host proteins (Shelton and Harris, 2008[102]). The interaction between the spike protein of SARS-CoV-2 receptor binding domain (RBD) and human angiotensin-converting enzyme 2 (ACE2) receptor is well documented (Lan et al., 2020[70]; Ridgway et al., 2023[95]; Zhou et al., 2020[121]). Recently, a study has shown that ORF8 of SARS-CoV-2 mimics IL-17 cytokines, contributing to severe inflammation during COVID-19 (Wu et al., 2022[116]). Domain Motif Interactions (DMI) are the primary mechanism of interaction between viruses and host proteins, where a single target molecule (SLiM) in the virus acts on a single target domain (host domain) (Halehalli and Nagarajaram, 2015[47]). DMI is an important therapeutic target. However, only a few published studies demonstrate the ability of DMIs to be drug targets. DMI targets are challenging due to their transient, heterogeneous, and complex nature. Another issue with DMIs is their physiological and structural properties. (Corbi-Verge and Kim, 2016[16]; Davey et al., 2011[18]). Only a few known DMIs are reported in the human proteome, showing that many are still to be discovered (Tompa et al., 2014[108]). In general, molecular mimicry is an exciting field of research to understand how viruses manipulate host cell routes and how viruses attack host cells (Goswami et al., 2023[43]). Ultimately, these discoveries will develop new antiviral therapeutic drugs (Corbi-Verge and Kim, 2016[16]; Davey et al., 2011[18]; Dyer et al., 2007[29]; Via et al., 2015[110]). Therefore, more advanced techniques (computational or experimental) are needed to study interactions based on SLiM, which is critical to understanding the mechanisms of motif mimicking in viruses (Evans et al., 2009[33]; Segura-Cabrera et al., 2013[101]).

## A General Overview of SLiMs

SLiMs are linear recurring peptides composed of 3-10 contiguous residues (Bhowmick et al., 2015[11]; D'haeseleer, 2006[23]; Davey et al., 2012[19]; Weatheritt et al., 2012[111]) often found in the disordered regions of proteins. SLiMs aid in mediating interactions with other partner proteins, although these interactions are transitory and have a low affinity of 1-150 μm (11-14 μm) (Diella et al., 2008[24]). SLiMs usually have only 2-5 defined positions and are challenging to detect by experimental and computational techniques (Neduva and Russell, 2005[81]). SLiMs play a role in various cellular pathways through DMIs, where rapid responses are required for transmission (Gibson, 2009[39]; Pancsa and Fuxreiter, 2012[89]). SLiMs can switch to multiple functionalities using a single-point mutation and are considered molecular switches. This plasticity of SLiMs gives pathogenic proteins an advantage, allowing them to mimic host proteins and aid in the host cell pathway (Dinkel et al., 2014[26]). To date, more than 4,138 new motif instances have been identified. While SLiM discovery is still in its early stages, there has been some progress in recent years. Various new computational tools and techniques have been developed to facilitate SLiM predictions of the protein sequence data. The significant repositories maintaining motif data include the eukaryotic linear motif database (ELM database) (Dinkel et al., 2012[25]; Kumar et al., 2020[68]), PROSITE (Hulo et al., 2006[54]), Linear Motif mediated Protein Interaction Database (LMPID) (Sarkar et al., 2015[99]), Minimotif-Miner (Balla et al., 2006[7]), PepCyber (Gong et al., 2008[42]) and Scansite (Obenauer et al., 2003[84]). The interaction of motif residues with domains suggests that these residues' positions will be conserved over time. Many SLiMs have two or more conserved hygroscopic residues, e.g., the nuclear export sequence (NES) has four (Gibson et al., 2015[40]). SLiMs are highly adaptable and act as molecular switches turned on or off by a single mutation. Such mutations may modify the function of the motifs. This property of SLiMs is being studied to design biological pathways that control different functions (Dueber et al., 2003[27]; Neduva and Russell, 2005[81]). For instance, a single TQG-to-TQT mutation can lead to synaptic transport in the neuron cells (Neduva and Russell, 2005[81]). The small size and polymorphic nature of SLiMs suggest that linear motifs are likely to have independent origins and can be used to find novel motifs that share interaction partners (Bhowmick et al., 2015[11]; Edwards and Palopoli, 2015[30]).

## High Throughput PPI Data as the Source for Predicting Motif Mimicry

Proteomic approaches have advanced during the past few years. These approaches play a significant role in identifying protein interactions, especially in understanding host-pathogen interactions. The current advancement in these methods has paved a new way to find novel PPIs. Different studies have discovered several novel interactions. The resulting interactions have been incorporated into several databases to help ease the process of further analysis (Lum and Cristea, 2016[75]). DMIs are used to detect new SLiMs. Unfortunately, most DMI information has been obtained from low-throughput studies (Blikstad and Ivarsson, 2015[12]). However, recently, various high throughput methods, including affinity purification in combination with mass spectrometry, yeast 2-hybrid and coimmunoprecipitations, have been employed to study DMI in multiple organisms (Mihalic et al., 2023[79]). These high throughput methods have enabled the generation of a large PPI dataset that is being used to predict functional DMIs and protein complexes. However, due to the high throughput nature of these high throughput experiments, false positives and false negatives are always present, impacting the chances of successful predictions (Li et al., 2010[71]; Zhang et al., 2015[120]). These high-throughput experiments have been conducted on various domain families, yielding a large amount of PPI data. Today, studies are being undertaken to determine SLiMs and related binding partners in human protein (Rajagopala et al., 2014[93]). 

Several methods have been used to study the SLiM-mediated interactions in viruses. Among these methods, two well-known methods are widely used to find vhPPIs. One of these methods is the Yeast two-hybrid (Y2H) method, which has produced a large amount of DMI data. This method splits a transcription factor binding domain and a DNA binding domain to bait or prey proteins. Through the interactions of the prey and bait proteins, the transcription factor leads to the activation of transcription of reporter genes. The advantage of using Y2H is its ability to study one protein or a library of proteins (Rolland et al., 2014[96]). Several successful studies have been carried out in recent years to identify DMIs. For example, SUMO interacting motifs have been discovered that interact with the SUMO1 and SUMO2 proteins (Hecker et al., 2006[51]). Y2H has not only successfully been used for the identification of DMIs but also for characterizing peptide binding motifs through screening peptide libraries. For example, Hu et al. have studied PDZ protein PDZK1 using Y2H screening against a random set of libraries (Hu et al., 2009[53]). Similarly, Guo et al. Investigated PDZ protein LNX using Y2H screening (Guo et al., 2012[46]). Y2H is used to find direct physical interactions between proteins, but most of the Y2H interaction data is transient and includes inter-complex interactions. The chances of false positives and false negatives are high, so Y2H might not be a precise source for binary interaction mapping (Zhang et al., 2015[119]). On the other hand, affinity purification followed by mass spectrometry (AP-MS) and Co-fractionation followed by MS (CoFrac-MS) is more suitable for identifying co-complex interactions, including direct and indirect interactions between proteins (Kim et al., 2010[63]). AP-MS works on purifying baits from cell lysate and detecting copurified proteins (the prey) by MS. The problem with AP-MS data is distinguishing between direct and indirect interactions (Luck et al., 2017[74]; Zhang et al., 2015[119]). CoFrac-MS is based on the fractionation of the protein extracts extensively to separate the protein complexes whose components are then detected by MS (Luck et al., 2017[74]). DMIs are currently underrepresented in available PPI networks and databases (Blikstad and Ivarsson, 2015[12]). During the past few years, these high throughput methods have been essential data sources for SLiM discovery by generating efficient PPI data. 

## Viral Mimicry by Different Viral Classes

Different computational approaches have been developed in recent years to study vhPPIs across the entire proteome (Dyer et al., 2007[29]; Evans et al., 2009[33]; Segura-Cabrera et al., 2013[101]), but most of these have focused on selected pathogens only (Barnes et al., 2016[9]; Chen et al., 2021[15]; Emamjomeh et al., 2014[32]; Zhang et al., 2017[118]). No study has been conducted to analyze different virus subtypes by their genetic makeup to understand how they disrupt the host cellular machinery responsible for regulatory functions and the infection cycle. So, it is interesting to know how different virus subtypes interact with the host proteins via SLiM. A few examples of experimentally validated mimicry candidates based on viral classes are given in Table 1(Tab. 1) (References in Table 1: Abada et al., 2008[1]; Accardi et al., 2011[2]; Ako-Adjei et al., 2015[3]; Bai and Nicot, 2012[5]; Barbera et al., 2006[8]; de Chassey et al., 2008[21]; Deng et al., 2005[22]; Falson et al., 2015[34]; Ganti et al., 2015[37]; Grzesik et al., 2019[45]; Han et al., 2004[48], 2020[49]; Incrocci et al., 2019[57]; Khan and Geiger, 2021;[62] Kirui et al., 2014[64]; Mechali et al., 2004[77]; Pal and Kundu, 2019[86]; Pan et al., 2018[88]; Ponnusamy et al., 2019[91]; Saridakis et al., 2005[98]; Segura-Cabrera et al., 2013[100]; Tavakolian et al., 2020[106]; Welcker and Clurman, 2005[112]; Zhang et al., 2018[117]).

### RNA viruses

RNA viruses are among the most harmful to human health and affect millions worldwide. RNA viruses are single-stranded or double-stranded and reproduce by using RNA-dependent RNA polymerases. A single-stranded virus, such as a retrovirus, infects a host cell with two copies of the single-stranded viral genome. The virus then undergoes reverse transcriptions to create viral DNA entering the host DNA. Viruses, including Ebola virus, HIV-1, Zika virus, Dengue virus, Influenza virus, Yellow fever virus, Adult Human T-cell lymphotropic virus type 1, Poliovirus, SARS virus and Retrovirus, are examples of RNA viruses (Poltronieri et al., 2015[90]). The RNA genome is responsible for generating viral proteins required for viral replication and some additional tasks, such as the template for the replication of genomic sequences, mRNA transcription and the assembly of virions. In some RNA viruses, the viral genome plays a crucial role in multiple processes in the host cell. Different viral and host enzymes interact with the virus genome to perform essential functions that help viruses replicate within host cells. In general, protein and various RNA factors interact with cell pathways to allow viruses to hijack host cell machinery (White et al., 2011[113]). In addition, RNA viruses have a high degree of structural and functional diversity. They can generate new RNA genomes at a rate of 0.4 s (provided the replication machinery is functioning optimally) (Moya et al., 2000[80]). It has been shown that viral hijacking through SLiMs is quite common in RNA viruses (Lieber et al., 2010[72]). Since there is currently no effective vaccine for most RNA viruses, it is essential to understand how the virus infects host cells and replicates by hijacking host cell machinery (Franzosa and Xia, 2011[36]).

### Single-stranded RNA viruses

There are two major categories of RNA single-stranded viruses: negative-stranded and positive-stranded. Negative-stranded viruses (NSV) are characterized by their genetic material consisting of a single strand of RNA.

**Figure 1:**
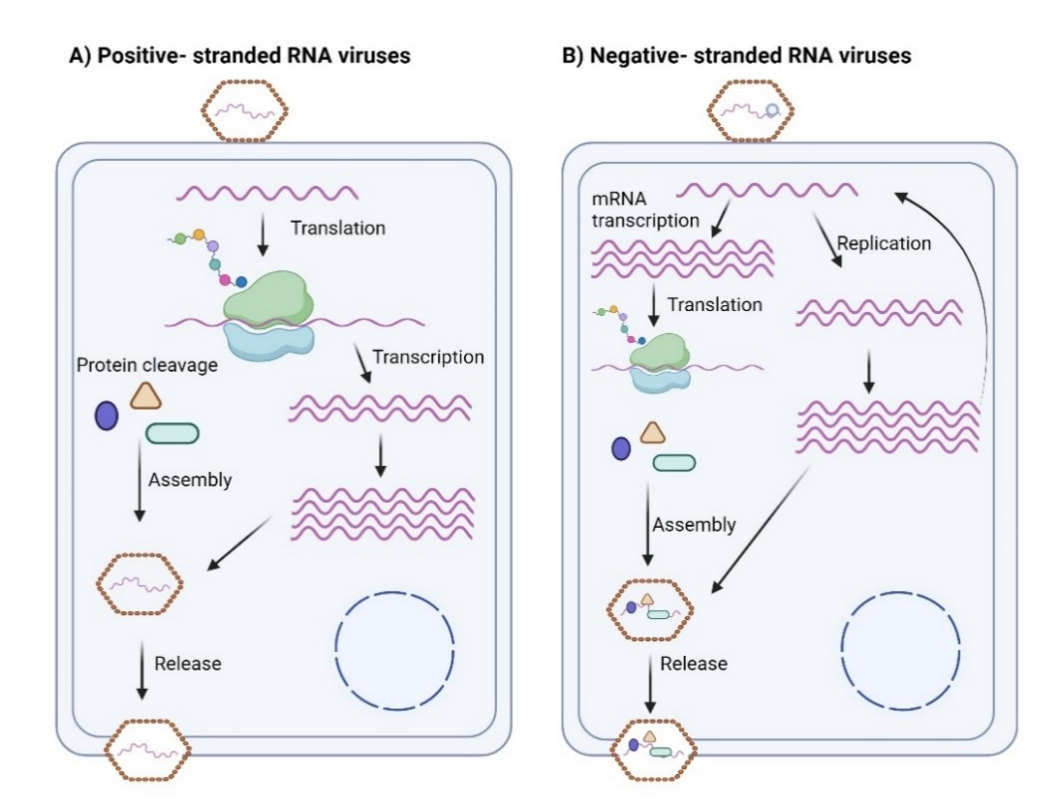
Replication cycle of ssRNA viruses. A) The replication cycle of PSVs: The virus is attached to a host cell and releases a +sense of its ssRNA. This +sense molecule is then translated into a single polyprotein molecule broken down into a protein. This protein is a template for the replication of the virus. B) The replication cycle of NSVs: The RNA in the NSV is transcribed into an mRNA, which can be further transcribed to a full-length +sense strand. The virus is then packaged and released from the cell, and the virus polymerase plays a role in transcription. Once the virus has been packaged and assembled, it is released outside of the cell (created using BioRender.com).

Positive-stranded virus (PSV) viruses are classified as segmented or non-segmented. Segmented viruses comprise families such as orthomyxovirus, Arenavirus, Bunyavirus, etc. Non-segmented viruses consist of families such as paramyxovirus, bornavirus, rhabdovirus, and filovirus. NSVs are characterized by their highly structured genome structures, which can be expressed as nucleocapsid or nucleoprotein complexes where genomic RNA is associated with more than one monomer nucleoprotein (nucleoside) (Green et al., 2014[44]; Ortin and Martin-Benito, 2015[85]). NSVs cause high mortality rates and have been implicated in many disease events, including influenza, measles, and mumps (Ortin and Martin-Benito, 2015[85]). NSVs begin their life cycle by attaching to a host cell, releasing their ssRNA. The released ssRNA is then translated into mRNA within the cell. mRNA is also transcribed to a genomic strand, which acts as a template for the virus genome replication. The transcription is done by a viral polymerase packaged inside the newly formed virion. Once the replicated virion has been assembled, it is released outside the cell (Figure 1B(Fig. 1)) (Stangler et al., 2007[104]).

PSVs, on the other hand, are an essential subclass of RNA viruses in which the RNA genome is plus-stranded. The RNA genome of a PSV serves as the template for the formation of viral proteins. It contains cis-acting RNA segments that regulate viral pathways such as viral replication, transcription, and translation (Liu et al., 2009[73]; Sztuba-Solinska et al., 2011[105]). During the life cycle of PSVs, the virus attaches to the host cell and releases the ssRNA into the cell. The ssRNA is translated into a polyprotein. Polysaccharides are further broken down into various proteins, including viral and RNA-dependent RNA polymerases. A complement of RNA is produced to serve as mRNA and aid in replicating the polysaccharide. Replicated information is assembled into a new virus and released outside the cell (Figure 1A(Fig. 1)) (Stangler et al., 2007[104]).

Generally, PSVs enter the host cell and replicate in its cytoplasm, where the host's immune system creates a hostile environment for viral replication. PSVs solve this problem by concentrating viral proteins in the intracellular space, allowing for continuous viral genome replication. PSVs hijack host factors that are involved in vascular transport and lipid synthesis. These host factors protect the virus replication machinery from the immune system, allowing for safe viral replication and assembly (Harak and Lohmann, 2015[50]). One of the most well-known examples of ssRNA virus motif mimicry is hepatitis C (HCV). HCV hijacks the host cell's machinery by replicating PxxP (known to bind to many SH3 domains in the Src kinases family) (Duro et al., 2015[28]). Another example of a PxxP motif is the hijacking of host cellular machinery in HIV by establishing interactions with the host SH3 domain (Stangler et al., 2007[104]).

### Double-stranded RNA viruses 

**Figure 2:**
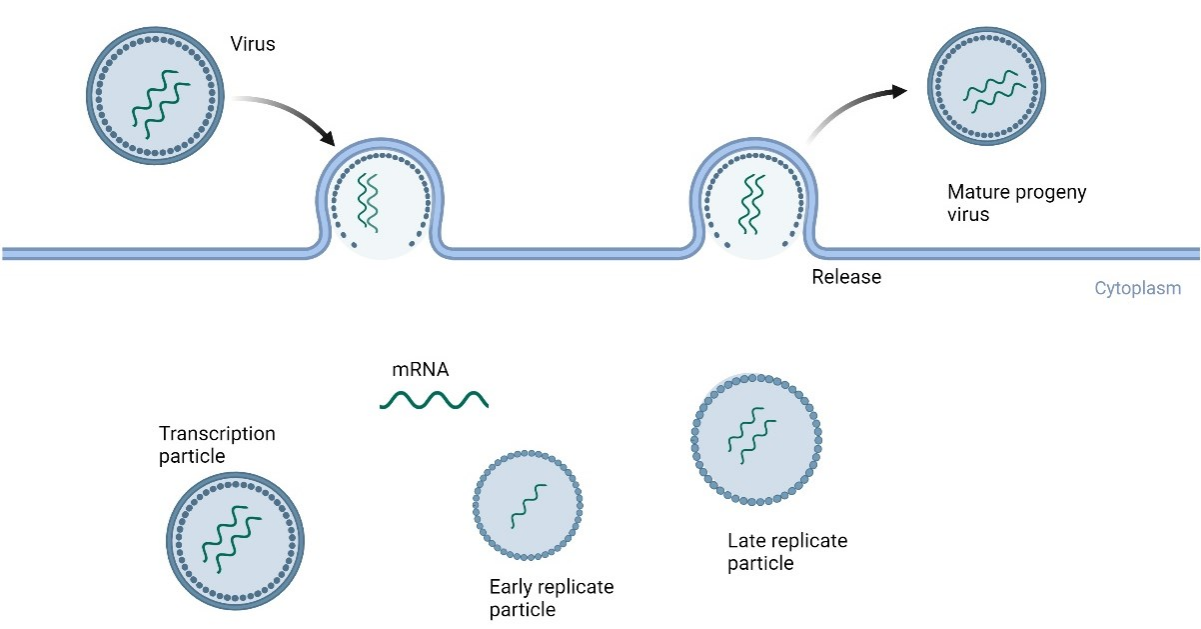
Replication cycle of dsRNA viruses. The virus attaches to the host cell and transcribes its dsRNA into mRNA. This mRNA is then packaged, resulting in the production of an early replicate particle. Subsequently, complementary RNA is produced, resulting in a late replicate particle released as a mature virus (created using BioRender.com).

Double-stranded RNA (dsRNA) is widely distributed in various organisms, including animal, plant, microbial, and non-molecular organisms-the first detection of dsRNA in a reovirus was in 1963 by Gomatos and Tamm (Wickner, 1993[114]). Most dsRNA viruses share similar capsid structures, replication strategies, and biochemical and structural characteristics. This is why cognate proteins of similar design and function can be identified from distantly related viruses, providing insight into their shared ancestry. It is well-known that dsRNA viruses replicate within the host cell's cytoplasm. These viruses enter the host cell and convert ssRNA into dsRNA. The genomic dsRNA then undergoes transcription into mRNA, which, upon translation, produces proteins necessary for viral replication (Figure 2(Fig. 2)). dsRNA viruses can replicate/transcribe their RNA within the icosahedral capsid due to the defense mechanisms of eukaryotic systems. These mechanisms detect and inactivate dsRNA through the presence of the inactivation protein PKR or the inactivation protein MDA5 (Mertens, 2004[78]). Furthermore, the viral proteins found in the internal virion-related enzymes and the innermost capsid layer are conserved mainly in most viruses. However, the non-structural proteins found in the outer capsid are diverse in sequence and structural organisation (Mertens, 2004[78]). A well-known example of dsRNA hijacking host cellular machinery is Segment-10 from Bluetongue, which hijacks host cell pathways by mimicking the functions of various host proteins (e.g., NEDD4, TSG101) (Wirblich et al., 2006[115]).

### DNA viruses

The prevalence and diversity of DNA viruses in eukaryotic organisms are significantly lower than that of RNA viruses. However, the emergence of giant viruses (with a genome size more significant than that of bacteria, archaea, and numerous parasitic unicellular organisms) has caused a shift in focus from RNA viruses to DNA viruses (Koonin et al., 2015[65]). DNA viruses are like RNA viruses, using a capsid to bind to and attack host cells (Ng et al., 2012[82]; Rao and Feiss, 2015[94]). Once they enter, they disassemble the virus and release the genome into the cell, transcribing it into mRNA. This mRNA is then translated into proteins, which help them hijack the host cells and make progeny viruses (Krupovic and Forterre, 2015[66]; Ng et al., 2012[82]). These progeny viruses are released outside the host cell, ready to attack other cells. There are two main types of DNA viruses - small (under 10 kb) and large (over 30 kb). Examples of small viruses include HPV and HBV, while giant viruses like Adenoviruses, Poxiviruses, and Herpesviruses are more common (Iyer et al., 2001[58]).

### Single-stranded DNA viruses 

**Figure 3:**
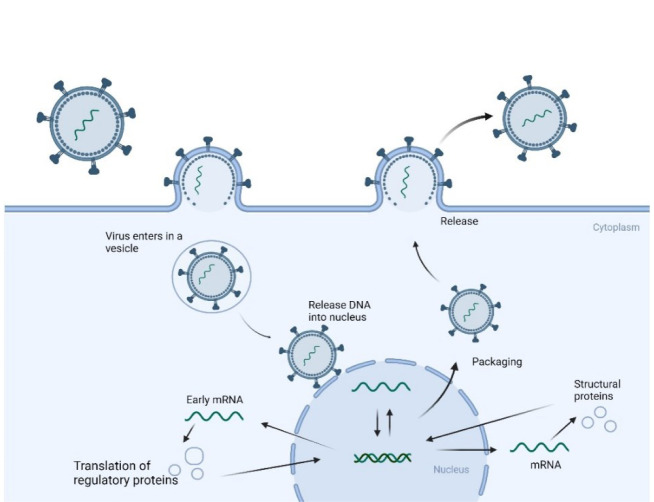
Replication cycle of ssDNA virus. Replication cycle of ssDNA virus. ssDNA viruses bind and enter their host cells to start their life cycle. As the ssDNA gets inside the vesicle, it releases its ssDNA within the nucleus—next, the ssDNA changes into dsDNA, which is then translated into early cytoplasmic mRNA. The early messenger RNA is then translated into different regulatory proteins that help to replicate the whole genome. Structural proteins are produced by translating the mRNA from the dsDNA transcription. Packaged and released outside the cell are newly duplicated ssDNA and structural proteins (created using BioRender.com).

These simple viruses have a single strand of DNA (ssDNA) as their genome. Most ssDNA viruses contain damaging strand DNA, but some include positive and negative strand DNA (Koonin et al., 2015[65]). ssDNA viruses have various invasion mechanisms depending on the host organisms (eukaryotes, bacteria, archaea, etc.). For example, the bacteriophage family Inoviridae have three different mechanisms of invasion of host cells. Some viruses utilize DDE transposase of IS30 family, IS3 family, IS110 family and IS492 family. Others encode the Serines/Tyrosin superfamily integrases and hijack the host recombinase mechanism. Eukaryotic ssDNA viruses integrate with host cells through the endonuclease function of their rolling ring replication initiation proteins (like bacteria transposon mechanisms) (Krupovic and Forterre, 2015[66]; Rosario et al., 2018[97]). These viruses contain one gene for coding the nucleocapsids in the virus and one for the DNA-encoding DNA replication enzyme. When the virus invades the host cell, it must convert its ssDNA genome to dsDNA, using DNA polymerase in the host cell to produce dsDNA. This dsDNA is then used as a template to transcribe the transcribed RNA into viral proteins. The replicated DNA is then converted to ssDNA, packaged, and reassembled into a new virus. Once packaged, the new virus is released outside the cell to infect new cells (Figure 3(Fig. 3)) (Krupovic and Forterre, 2015[66]).

### Double-stranded DNA viruses 

**Figure 4:**
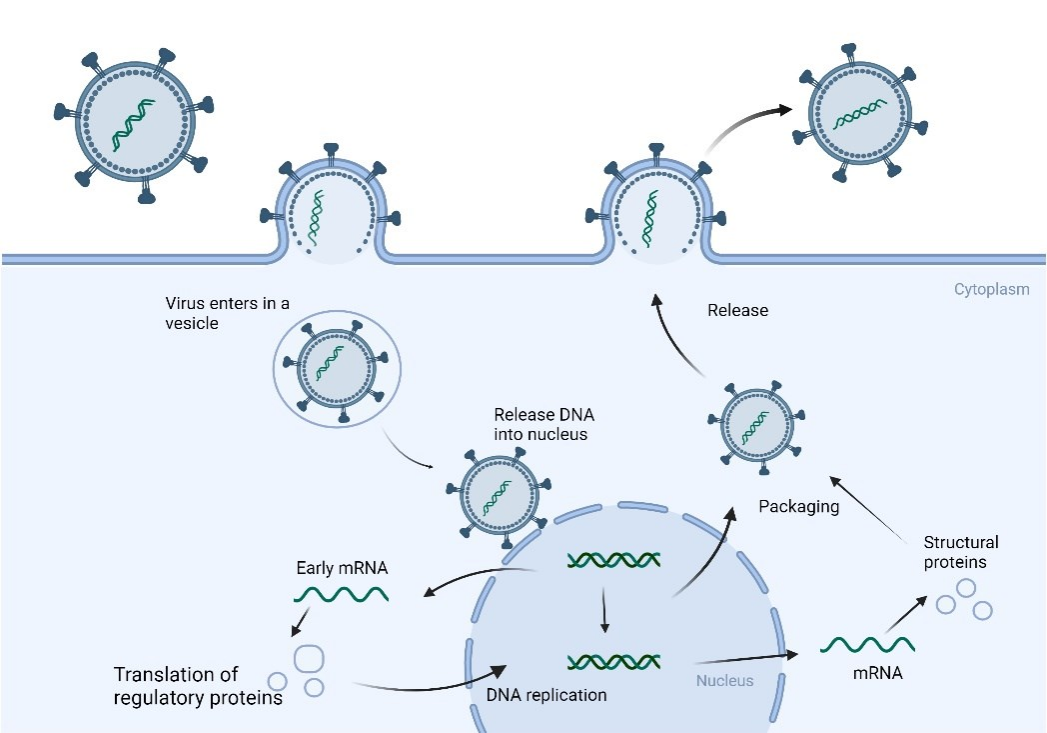
Replication cycle of dsDNA virus. The attachment and penetration of the virus into the cell marks the start of the dsDNA virus life cycle. A vesicle carrying the virus enters the cell. Once it enters the nucleus, it releases its dsDNA molecule. The dsDNA gets translated into mRNA once it is within the nucleus. Regulatory proteins are produced through translation of the mRNA in the cytoplasm. Regulatory proteins aid DNA replication and mRNA transcription into structural proteins. After being enclosed in a capsid, the freshly copied DNA and the structural proteins are liberated from the cell (created using BioRender.com).

A double-stranded DNA (dsDNA) virus has a single molecule dsDNA as its genome (Wickner, 1993[114]). Many dsDNA virus families infect mammals, such as Hepadnavirus, Papillomirus, Polyomirus, Herpesvirus, Adenovirus, Asfarvirus, and Poxvirus (Koonin et al., 2015[65]). All these viruses, except Asfarvirus, infect humans or animals. dsDNA viruses are often considered the simplest to understand regarding their life cycle. The life cycle of a dsDNA virus starts when a virus attacks the host cell and viral DNA enters the host cell's nucleus, replicating the host genome. Host cell DNA polymerase replicates viral DNA, and then the mRNA is transcribed into various viral proteins. Some of these proteins are capsids in which newly replicated dsDNA is packaged. Once packaged, the packaged virion is released outside the cell to infect other cells (Figure 4(Fig. 4)) (Kazlauskas et al., 2016[60]; Kazlauskas and Venclovas, 2011[61]; Rao and Feiss, 2015[94]). A well-known example of dsRNA motif mimicry is the PxLTXP motif in the HADC serotype 5 E1 protein interacting with the human BS69 protein MYND domain. Hijacking aids viruses in controlling their viral replication at the time of infection (Zhang et al., 2018[117]). Another example of dsDNA virus motif mimicry is where the PDZ-binding motif in the HPDC E6 protein targets host cell PDZ-containing proteins (Accardi et al., 2011[2]; Segura-Cabrera et al., 2013[100]).

## SLiM-Mediated Prediction of Viral Mimicry

Understanding the functional importance of SLiMs and clarifying their roles in different biological pathways requires computational prediction of these motifs. Various computational SLiM discovery tools have been developed in the post-genomic era to help find putative SLiMs in protein sequences and expand our knowledge of molecular relationships and cellular signalling networks (Idrees et al., 2018[56]). Several SLiM discovery tools have been developed; a few are listed in Table 2(Tab. 2) (References in Table 2: Bailey et al., 2015[6]; de Castro et al., 2006[20]; Jones and Cozzetto, 2015[59]; Krystkowiak and Davey, 2017[67]; Kumar et al., 2022[69]; Palopoli et al., 2015[87]; Prytuliak et al., 2017[92]). Successful prediction of SLiMs using computational tools can also help develop antiviral drugs (Simonetti et al., 2023[103]). The general criteria in such cases are to find SLiMs in viral proteins involved in virus replication, entry, or defence evasion (Davey et al., 2012[17]). Once essential SLiMs have been identified, the next step is to create therapeutic agents (e.g., small molecules, peptides) that imitate or interfere with these SLiMs. This strategy breaks viral protein chains and disrupts viral functions (Mahajan et al., 2021[76]). Most viruses use specific SLiMs to enter host cells; therefore, antiviral methods that block viral entry motifs can help prevent the virus from infecting the cells. Moreover, small peptide-based inhibitors can help interfere with SLiM-mediated interactions, disrupting viral replication and slowing the infection (Bah and Forman-Kay, 2016[4]). 

In general, computational prediction of SLiM-mediated viral mimicry rapidly progresses, revealing viruses' complex interactions with their hosts. These relationships are necessary for pathogenesis and viral replication. Researchers using advanced computational resources and techniques are finding ways to emulate viral campaigns. This data has the potential to generate novel antiviral therapies in addition to deepening our understanding of vhPPIs. Moreover, developing antiviral strategies using SLiMs as targets is a multi-faceted approach that involves understanding the intricacies of vhPPIs and designing interventions that disrupt these interactions. It's a promising avenue in the battle against viral infections, and continued research in this field may yield innovative antiviral therapies that are effective and less prone to resistance development (Bah and Forman-Kay, 2016[4]).

## Conclusion

Viruses enter the host cell and start their replication process to propagate. Due to their restricted genome size, viruses have evolved to utilize host cellular machinery to continue their life cycle and escape the host defense system. Viruses use short sequences of linear motifs to mimic and hijack host cellular proteins. Therefore, it's essential to understand the short linear motif-mediated interactions between viruses and their host proteins. This review covers the current knowledge of viral mimicry and gaps in knowledge that will be important for future studies to investigate.

## Declaration

### Conflict of interest 

The authors declare that they have no conflict of interest.

### Acknowledgments

The authors thank the University of New South Wales, Sydney, and Dr. Richard J. Edwards. 

### Author contributions

SI wrote the manuscript. KRP, and PMH helped in revision. TS helped in making figures.

### Footnotes

This review article contains partial excerpts from Idrees' dissertation (2020[55]).

## Figures and Tables

**Table 1:**
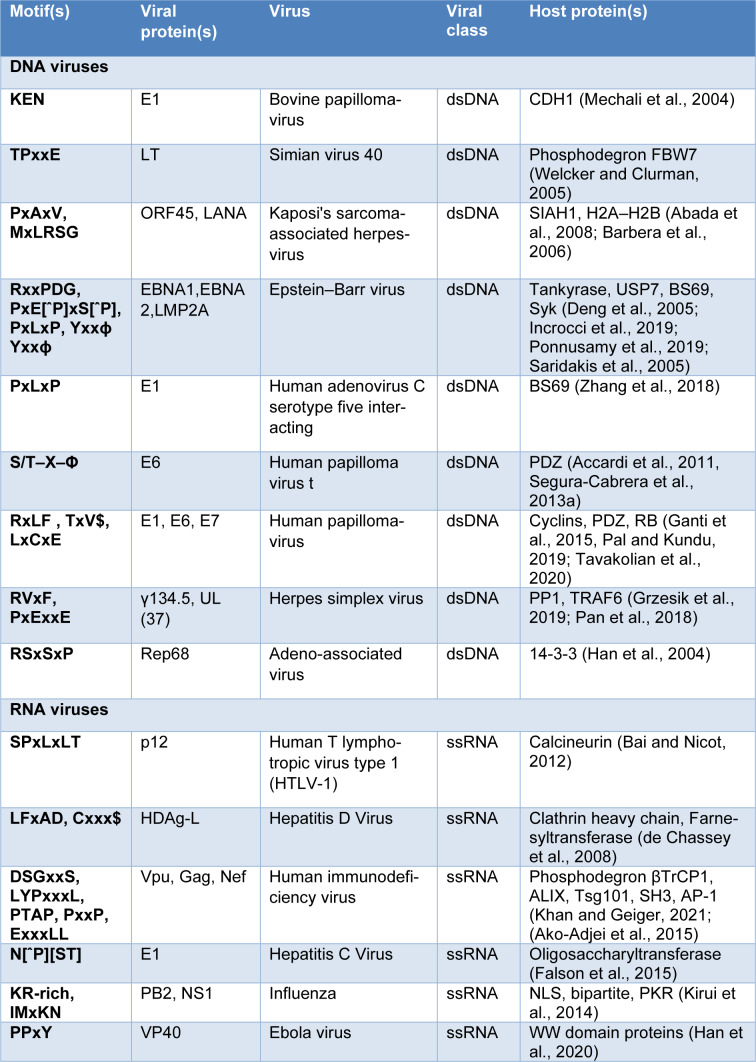
Examples of viral mimicry candidates characterised by viral subtypes

**Table 2:**
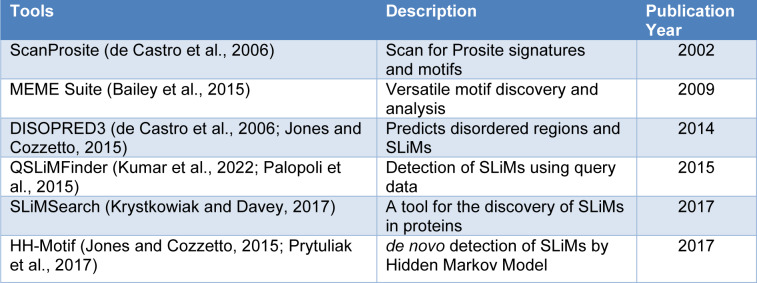
SLiM discovery/prediction tools that can help in predicting new mimicry candidates
